# Dl-3-n-Butylphthalide Alleviates Demyelination and Improves Cognitive Function by Promoting Mitochondrial Dynamics in White Matter Lesions

**DOI:** 10.3389/fnagi.2021.632374

**Published:** 2021-03-08

**Authors:** Yiwei Feng, Min Guo, Hongchen Zhao, Sida Han, Yining Hao, Yiwen Yuan, Weiwei Shen, Jian Sun, Qiang Dong, Mei Cui

**Affiliations:** ^1^Department of Neurology, Huashan Hospital, Fudan University, Shanghai, China; ^2^Department of Neurology, Huashan Hospital, State Key Laboratory of Medical Neurobiology and Ministry of Education Frontiers Center for Brain Science, Fudan University, Shanghai, China

**Keywords:** Dl-3-n-butylphthalide, white matter lesions, mitochondria dynamics, demyelination, cognitive impairment

## Abstract

White matter lesions (WMLs) are a type of cerebrovascular disorder accompanied by demyelination and cognitive decline. Dl-3-n-butylphthalide (D1-NBP) is a neuroprotective drug used for the treatment of ischemic cerebrovascular diseases, although the function of DI-NBP on WML is still not clear. This study aims to investigate whether DI-NBP affects cognitive function and ameliorates demyelination in a model of WML. The bilateral carotid artery stenosis (BCAS) mouse model and *in vitro* brain slice cultures with low glucose and low oxygen (LGLO) treatment were adopted. The Dl-NBP was administered intragastrically for 28 days after BCAS or added at a dose of 50 μm for 48 h after LGLO. Spatial learning and memory were evaluated by an eight-arm radial maze. Demyelination was detected using a TEM. Mitochondrial dynamics were assessed by time-lapse imaging in the cultured brain slices. The function of the synapse was evaluated by the patch clamp technique. In BCAS mice, obvious demyelination and cognitive decline were observed, while both were significantly relieved by a high-dose D1-NBP treatment (100 mg/kg). Along with demyelination, mitochondrial accumulation in the axons was significantly increased in the BCAS mice model, but with the treatment of a high-dose D1-NBP, mitochondrial accumulation was mitigated, and the anterograde/retrograde transport of mitochondria was increased. Following the improved anterograde/retrograde transport of mitochondria, the synapse activity was significantly upregulated while the reactive oxygen species (ROS) generation was remarkably decreased in the cultured brain slices. In addition, we identified syntaphilin (SNPH) as the downstream target of D1-NBP. The overexpression of SNPH mediated the effects of D1-NBP in mitigating axonal mitochondrial accumulation. In conclusion, the D1-NBP treatment significantly relieved demyelination and improved spatial learning and memory in the WML model by promoting mitochondrial dynamics. These neuroprotective effects of D1-NBP were mediated by inhibiting the mitochondrial arching protein, SNPH, which provided a potential therapeutic target for WML.

## Introduction

White matter lesions (WMLs) are one of the major contributors that lead to cognitive decline and vascular dementia (VaD), especially among the elderly (Alber et al., 2019). The WML is usually caused by a modest, but chronic, reduction of blood flow, accompanied by a shortage of oxygen supply through small vessels (Ben-Ari et al., 2019). Demyelination is a characteristic of the pathological changes in WML, which accounts for the worse clinical outcomes and impaired cognitive function in patients (Datta et al., 2017). It is, therefore, urgent to figure out how to alleviate the demyelination damage and mitigate the impaired brain function.

In 2002, the FDA of China approved the use of Dl-3-n-butylphthalide (Dl-NBP), a compound extracted from the seeds of celery in treating ischemic stroke (Wang et al., 2018; Chen et al., 2019; Yang et al., 2019). It is also undergoing a Phase II trial for the treatment of ischemic stroke in the USA (Cui et al., 2013; Xue et al., 2016). Previously, more attention was given to the acute phase of stroke, and the neuroprotective effects of Dl-NBP on stroke are supported widely by both clinical and basic research (Chen et al., 2020). Mechanically, studies have shown that the Dl-NBP could inhibit the apoptosis of neurons, endoplasmic reticulum stress, and oxidative stress and improve hemodynamics as well as neurogenesis (Sun et al., 2017; Wang et al., 2018, 2019). However, as a promising molecular compound in treating ischemic injuries of the brain, the function of Dl-NBP in WML and demyelination is neither clear nor has it been confirmed.

From the limited studies on WML, evidence shows that the Dl-NBP could promote the cognitive function in VaD models caused by chronic hypoperfusion (Li et al., 2019). The normal cognitive function requires a relatively normal electronic signal and a synaptic signal, which rely on intact myelin, as well as cholinergic neurotransmission (Feng et al., 2020b). The beneficial effects of Dl-NBP on VaD were demonstrated by improving remyelination and enhancing the function of the cholinergic system (Tian et al., 2020). Yet, remyelination happens at the late stage of WML injury and the function of the cholinergic system is closely related to the electronic signal from the axons (Lema et al., 2017). Whether the Dl-NBP can benefit the electronic function of synapse or alleviate myelin breakdown at the early stage remains to be determined.

Under hypoperfusion, mitochondria are the most sensitive organisms in neurons that sense ischemia/hypoxia and quickly change their dynamics and metabolism (Bargiela et al., 2018). Axons suffer ischemic damage and display abnormal mitochondrial dynamics, showing disturbed fission–fusion transport in axons (Chen et al., 2018; Thomas and Ashcroft, 2019). The reduced retrograde transport of dysfunctional mitochondria can block mitophagy and cause unfavorable reactive oxygen species (ROS), which is harmful to myelin (Palikaras et al., 2015). The anterograde of mitochondria is vital for the supply of energy to the synapse for neurotransmitter release (Zheng et al., 2019). Therefore, we speculated that the Dl-NBP might relive demyelination in WMLs by regulating mitochondrial dynamics. In the present study, we investigated the effects of Dl-NBP on mitochondrial dynamics and demyelination using a WML model.

## Materials and Methods

### Animals

C57BL/6J male mice (9–12 weeks, 25–30 g) were purchased from Charles River Laboratories and housed in the Experimental Animal Center of Fudan University, Shanghai, China in a temperature- and humidity-controlled specific-pathogen-free laboratory with a 12/12 h light/dark cycle. All procedures were performed in accordance with the Guide for the National Science Council of the People's Republic of China, and the study was approved by the Ethics Committee of Fudan University (IRB approval number 20190972A259). This manuscript was written in accordance with the Animal Research: Reporting of *in vivo* Experiments (ARRIVE) guidelines.

### The BCAS Model and DI-NBP Treatment

The bilateral carotid artery stenosis (BCAS) model was performed as described previously (Feng et al., 2020b). Briefly, the mice were anesthetized using 4% isoflurane in 28% O_2_ and 68% N_2_ and maintained on 2% isoflurane in 29% O_2_ and 69% N_2_ by a mask. After making a midline skin incision on the neck, the bilateral common carotid arteries were isolated and subsequently stenosed using 0.18 mm steel micro coils (Wuxi Samini/Sawane Spring Co., Ltd., Hamamatsu City, Japan). For sham-operated mice, a similar procedure was followed, whereas micro-coils were not used for the induction of BCAS. For the D1-NBP treatment, a low-dose treatment of DI-NBP (L-NBP, 50 mg/kg/day) and a high-dose treatment of D1-NBP (H-NBP, 100 mg/kg/day) were intragastrically administrated 1 day after the BCAS surgery for 28 days.

All experimental groups were randomized, and all outcome analyses were carried out by independent investigators blinded to the treatment conditions and mouse types. Randomization of each experimental group was performed before the surgical procedure by using the random number generator in GraphPad. The preliminary data from the TEM and the eight-arm maze experiments indicated that 6 and 12 animals per group, respectively, would be sufficient to obtain 80% power at a significance level of < 0.05 with a two-sided test.

### The Cerebellum Slice Culture and the D1-NBP Treatment

For cerebellar organotypic slice culture, the postnatal day 8–9 (P8-9) mice were used. About 400 μm P8-9 mouse cerebellum parasagittal slices were obtained using a vibratome (ZQP-86, Zhixin Co., Ltd., Shanghai, China). The slices were placed on cell culture inserts (Millipore, Bedford, MA, USA) and were cultured in 50% Dulbecco's modified eagle's medium (DMEM) with 25% Hanks' balanced salt solution (HBSS), 25% horse serum, and 5 mg/ml glucose (Invitrogen, Carlsbad, CA, USA) in cell culture chambers at 37°C.

The low glucose and low oxygen (LGLO) (2% O_2_ and 1 mg/ml glucose) treatment was used to give a chronic hypoperfusion environment to the slices for 48 h. The D1-NBP, at a dose of 50 μm, was added to the cultured slices before the LGLO treatment, which is a relatively high dose consistently used with previous studies (Li et al., 2019). Experiments and data analyses were performed in a double-blinded manner.

### The Overexpression of Syntaphilin

The overexpression (OE) of syntaphilin (SNPH) plasmid and adeno-associated virus (AAV) 2/9 or lentivirus was constructed by Genomeditech (Genomeditech, Shanghai, China). For culturing cerebellum slices, lentivirus was added to the medium of the slice at 1^*^10^10^ gene copies, 5 days before the LGLO treatment.

For mice, AAV 2/9 SNPH-OE plasmid or AAV empty vector was stereoscopically injected into lateral ventricles. The mice were anesthetized using 4% isoflurane in 30% O_2_ and 70% N_2_ and maintained on 2% isoflurane in 30% O_2_ and 70% N_2_ by a mask. The AAV vectors were infused into the left lateral ventricle (coordinates from bregma: AP, −0.2 mm; ML, +1.0 mm; DV, −2.3 mm). The genome copies of size 5 × 10^11^ were infused at a rate of 1 μl/min. After injection, the needle was left in place for 2 min to prevent backflow before the withdrawl.

### Time-Lapse Imaging Using Confocal Microscopy

As described previously (Lin et al., 2017), the mitochondria were labeled with MitoTracker Red CMXRos, M7512 (Thermofisher Scientific, USA) for 3 h after the brain slices were treated with LGLO. After an extensive wash, the slices were placed in an airstream incubator at 37°C and imaged by an Olympus inverted confocal microscope using a 60 × 1.3 NA oil immersion objective with 512 × 512-pixel resolution (FV1200, Olympus).

Upon imaging, a total of 5 min with 15 s intervals were imaged for each experiment. The total live imaging time was restricted to 20 min to minimize phototoxic damage. The length, area, and diameter of the axonal mitochondria were measured by the ImageJ program (NIH, USA). The number and mean velocity of motile mitochondria were analyzed by kymographs. Stationary sites in this study were defined as CMXRos-positive profiles that were stationary during a 5-min period. To measure the size of the stationary mitochondria, a pair of image stacks, including all CMXRos-positive profiles of each axon, were obtained at the time periods 0 and 5 min.

### Immunofluorescence

Brain slices were fixed overnight in 4% paraformaldehyde (PFA) and then in 30% sucrose for 2 days at 4°C. Subsequently, the slices were blocked with 5% bovine serum albumin (BSA) for 1 h and permeabilized with 0.1% Triton X-100 in phosphate buffered saline (PBS) for 15 min. Primary antibodies, diluted in a blocking buffer, were added to the slices and were incubated overnight at 4°C. The primary antibodies used in this experiment were anti-NF (1:50, ab8135, Abcam, USA) and anti-maltose binding protein (MBP) (1:200, ab40390, Abcam, USA). The slices were washed three times with PBS and labeled with a fluorescence-conjugated secondary antibody for 1 h at room temperature (Alexa Fluor 488 and 594, 1:1,000, Life Technologies). Nuclei were visualized by mounting with DAPI (28718-90-3; Sigma Aldrich, USA).

For ROS staining in mitochondria, the MitoSOX™ Red mitochondrial superoxide indicator (M36008, ThermoFisher, USA) was used to label ROS in mitochondria. The MitoSOX was diluted according to the manufacturer's instruction and incubated with the slices for 3 h. After an extensive wash with PBS, the slices were replenished with the indicated culture medium.

### Western Blot

Brain slices were collected and lysed in the radioimmunoprecipitation assay (RIPA) buffer [50 mm Tris-HCl, pH 7.5, 150 mm NaCl, 1% Triton X-100, 0.1% sodium dodecyl sulfate (SDS), 0.5% deoxycholate] with a protease inhibitor. Equal amounts of proteins, measured by the BCA method, were loaded on 15% Bris-Tris NuPAGE, electrophoresed, and transferred into 0.22 μm nitrocellulose membranes. After blocking with 5% BSA in TBST for 1 h, the membranes were incubated overnight at 4°C with the following primary antibodies: anti-SNPH (ab69992, Abcam, USA), anti-Miro1 (ab188029, Abcam, USA), anti-Trak1 (ab28751, Abcam, USA), anti-HSP60 (ab190828, Abcam, USA), and anti-β-actin (ab115777, Abcam, USA) at a dilution of 1:1,000 (Tris-buffered saline with 0.1% Tween^®^ 20 detergent).

### Transmission Electron Microscope

The TEM was performed as described previously (Guo et al., 2019). In short, the brain samples were perfused with PBS and 4% paraformaldehyde (PFA). Dissected tissues (1 mm in thickness) were postfixed in buffered OsO_4_, dehydrated in graded alcohol solutions and propylene, embedded in Epon, and examined by light microscopy after staining with toluidine blue. Thin sections cut on using formvar-coated slot grids and stained with uranyl acetate and lead citrate were examined using a JEOL 1200 electron microscope. G-ratios were determined as the inner to outer axonal circumference ratio using the ImageJ program.

### The Eight-Arm Radial Maze Test

The eight-arm radial maze test was performed as described previously (Xu et al., 2019). The maze consisted of a central platform (24 cm in diameter) with eight arms that extended radially. The mouse was allowed to visit each arm to eat eight pellets in food cups placed near the end of each arm. Each test animal was trained once per day to memorize the apparatus. The performance of the test animals in each trial was assessed using the two parameters, namely the number of correct choices in the initial eight chosen arms and the number of errors (defined as choosing arms that had already been visited). When the test animals had made seven or eight correct choices with no more than one error in three successive sessions, they were deemed to have memorized the maze.

### Whole-Cell Patch-Clamp Electrophysiology

As described previously (Feng et al., 2020a), the cultured brain slices were transferred to the patch-clamp bath solution for 1 h prior to recording. The bath solution contained 126 mm NaCl, 2.5 mm KCl, 26 mm NaHCO_3_, 1.25 mm Na_H_2_*PO*4_, 2 mm CaCl_2_, 2 mm MgCl_2_, and 11 mm glucose bubbled with 95% *O*_2_ + 5% CO_2_. The temperature of the bath solution was maintained at 32°C. For miniature excitatory postsynaptic current (mEPSC) recordings, patch pipettes containing 126 mm K-gluconate, 4 mm KCl, 4 mm ATP-Mg, 0.3 mm GTP-*Na*_2_, 10 mm PO creatine, 10 mm HEPES, and 0.2–0.5% biocytin (pH 7.3 adjusted using KOH, 300 mOsm maintained using sucrose) with a tip resistance of 6–8 MΩ were used. During mEPSC recording, tetrodotoxin (TTX) (0.5 μm) was administered to silence the network activity through the inhibition of voltage-sensitive sodium channels, and bicuculline (10 μm) was given to block the GABA-A-mediated inhibitory signaling.

For the recording of action potentials (APs), patch pipettes containing 140 mm K-gluconate, 5 mm ethylene glycol-bis(β-aminoethyl ether) (EGTA), 0.5 mm CaCl_2_, 2 mm ATP-Mg, 0.3 mm guanosine-5′-triphosphate (GTP)-Na_2_, 10 mm sucrose, 10 mm HEPES, and 0.2–0.5% biocytin (pH 7.3 adjusted using KOH, 300 mOsm maintained using sucrose) with a tip resistance of 6–8 MΩ were used.

Series resistance was monitored at an interval of 2 min, and recordings were excluded if the series resistance and leak current changed significantly and/or exceeded 40 MΩ or 200 pA, respectively.

### Golgi Silver Staining

Golgi silver staining was performed as described previously (Du, 2019). The mice were sacrificed and perfused with 4% PFA. The brain was dissected, cut into half at the junction between the cortex and midbrain, and further incubated in the PFA solution for a further 10 min, followed by the immersion in the Golgi solution (FD Neurotechnologies, Rapid Golgi Kit). The Golgi solution was changed after 6 h, and the brain was kept immersed as such for 2 weeks before development as per the instructions of the manufacturer.

### Statistical Analysis

Data were analyzed using SPSS Statistics 22 and graphed with GraphPad Prism 8.0. The sample size was calculated based on the power as 0.95 and α as 0.05. All data from the mice are represented as the mean ± SD and the data from the brain slices are represented as mean ± SEM. Different treatment groups were evaluated using a one-way ANOVA with the Tukey's test for multiple comparisons. The null hypothesis was rejected when *p*-value was < 0.05.

## Results

### DI-NBP Mitigates Demyelination and Improved Cognitive Impairment in the BCAS Model

**Figure 1:**
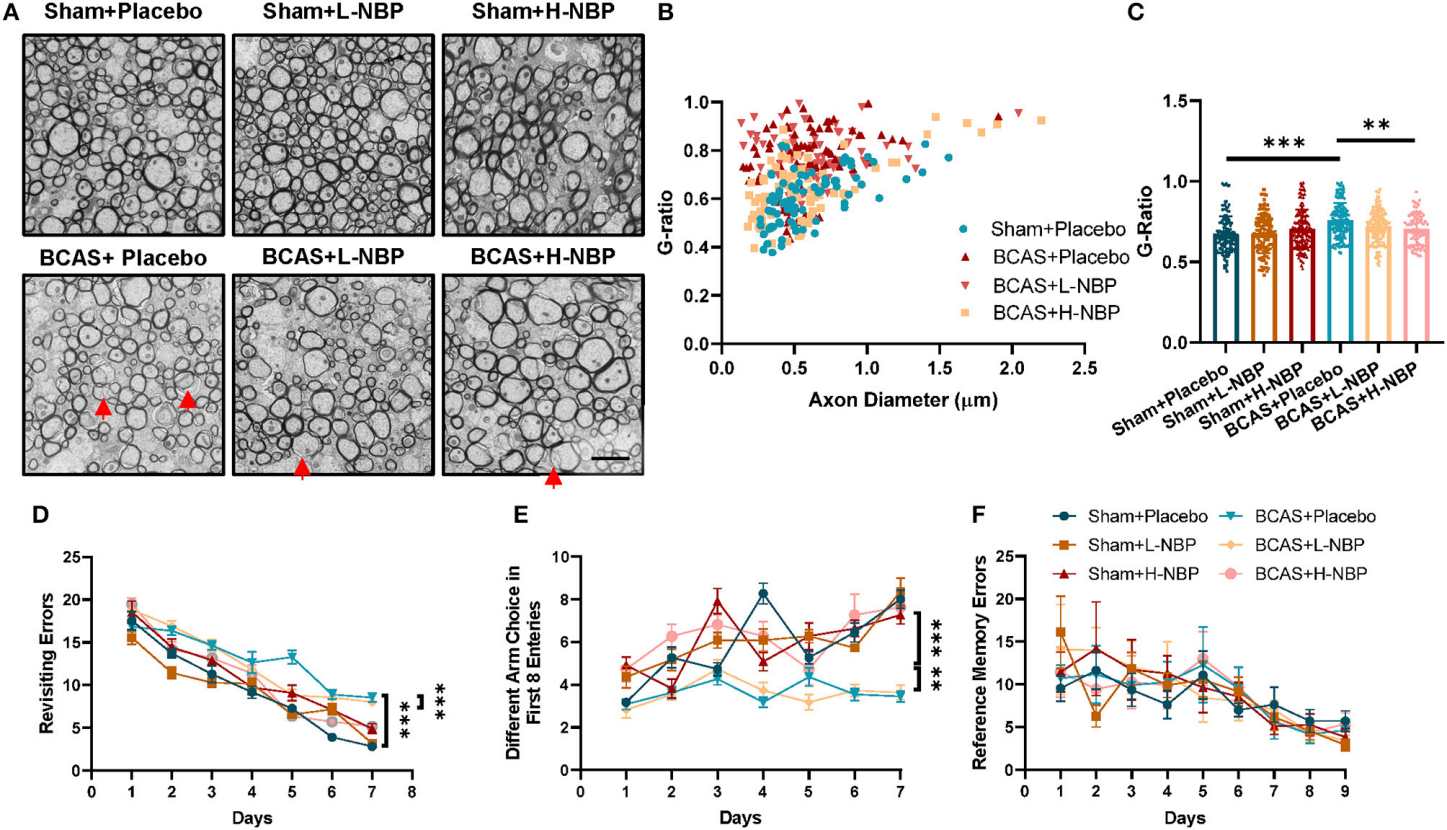
**(A)** High-dose treatment of Dl-3-n-butylphthalide (D1-NBP) mitigated demyelination and improved the cognitive impairment in the bilateral carotid artery stenosis (BCAS) model. **(A–C)** Representative TEM images **(A)** and quantitative analysis of the G-ratio **(B,C)** in Sham+Placebo, Sham+L-NBP, Sham+H-NBP, BCAS+Placebo, BCAS+L-NBP, and BCAS+H-NBP groups. Scale bar, 2 μm. A one-way ANOVA with Tukey's correction. *n* = 80 myelinated axons (20 axons per mouse, 4 mice per group). **(D–F)** Working and reference memory were assessed by the eight-arm radial maze. Impaired working memory in the BCAS mice was remitted by a high-dose administration of DI-NBP, as evidenced by the less revisiting errors **(D)** and more different choices **(E)** in the BCAS+H-NBP group. There is no significant difference in the spatial reference memory between different groups **(F)**. A two-way ANOVA with the Dunnett's *post-hoc* test, *n* = 11 mice in each group. Data are represented as means ± SD (^**^p < 0.01; ^***^p < 0.001; ns: non-significant differences). L-NBP, low dose of DI-NBP; H-NBP, high dose of D1-NBP.

To study the therapeutic potential of D1-NBP in mitigating demyelination and cognitive impairment caused by whole-brain hypoperfusion, we used the BCAS mouse model in which coils were placed around the bilateral common carotid arteries. After BCAS modeling, demyelination and cognitive impairment were significant at day 28 after BCAS, indicating the successful modeling of hypoperfusion-induced VaD (Figures 1A–F). For the D1-NBP treatment group, the mice were given daily intragastric administration of Dl-NBP or Placebo 1 day after BCAS until sacrifice. The status of myelination of the different treatment groups was evaluated by TEM. It could be observed that the whole-brain hypoperfusion by BCAS changed the overall axonal G-ratio distribution to a higher G-ratio rate, which is accompanied by a significant increase of G-ratio in the BCAS group. Although a low-dose treatment of D1-NBP (L-NBP, 50 mg/kg/day) did not retrieve demyelination, an increased dose of D1-NBP treatment (H-NBP) to a 100 mg/kg/day robustly alleviated the BCAS-induced demyelination (Figures 1A–C).

We further evaluated the therapeutic effect of D1-NBP on BCAS-induced cognitive impairment, which is tested by the eight-arm radial maze. In the BCAS treatment group, the mice exhibited higher revisiting errors and a lower different arm choice in the first eight entries, indicating a significant impairment of working memory in the BCAS mice. Reference memory errors showed that reference memory was not influenced by BCAS. Consistent with the previous results, the high dose DI-NBP treatment significantly mitigated the impaired working memory by BCAS (Figures 1D–F). Altogether, we found that a high-dose treatment of DI-NBP enabled the retrieval of demyelination and cognitive impairment induced by whole-brain hypoperfusion.

### Alleviated Demyelination and Cognitive Impairment by DI-NBP Treatment Is Accompanied With Decreased Mitochondrial Accumulation Among the Axons

**Figure 2:**
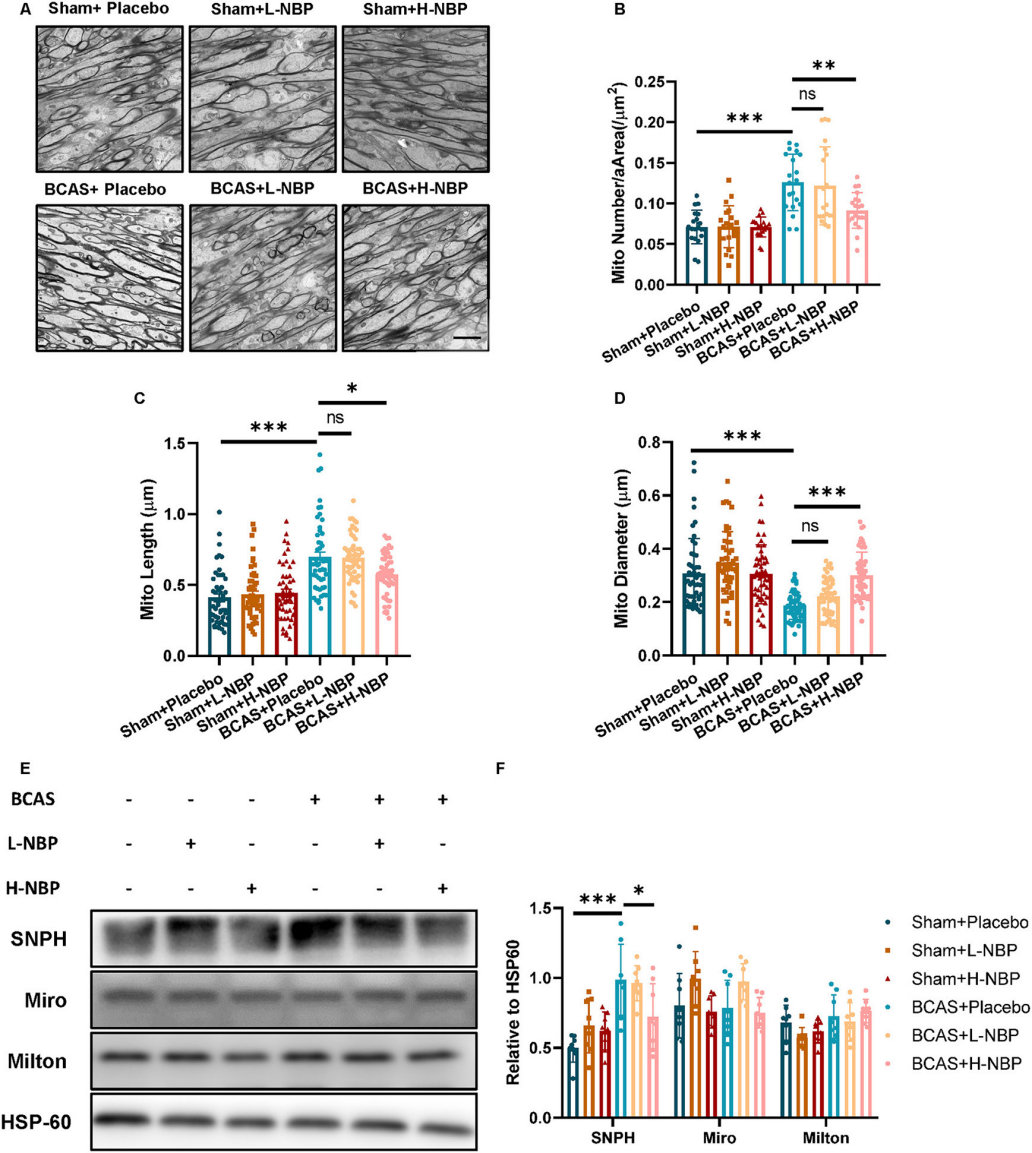
Alleviated demyelination and cognitive impairment by a high-dose treatment of D1-NBP was accompanied by decreased mitochondrial accumulation among the axons. **(A–D)** Representative TEM images of mitochondrial load in axons **(A)** and quantitative analysis of mitochondrial number per area (/μm^2^) **(B)**, mitochondrial length **(C)** and mitochondrial diameter **(D)** in Sham+Placebo, Sham+L-NBP, Sham+H-NBP, BCAS+Placebo, BCAS+L-NBP, BCAS+H-NBP groups. Scale bar, 2 μm. A one-way ANOVA with Tukey's correction. For mitochondrial load per area analysis, *n* = 20 visual fields (4 visual fields per mouse, 5 mice per group). For mitochondrial length analysis, *n* = 50 (10 mitochondria per mouse, 5 mice per group). For mitochondrial diameter analysis, *n* = 50 (10 mitochondria per mouse, 5 mice per group). **(E,F)** Immunoblot **(E)** and quantitative analysis **(F)** of syntaphilin (SNPH), Miro1, and Milton in different groups. A one-way ANOVA with Tukey's correction, *n* = 8 mice per group. Data are represented as means ± SD (^*^p < 0.05; ^**^p < 0.01; ^***^p < 0.001; ns, non-significant differences).

Compared to myelin, the axons are more vulnerable to the hypoxic-ischemic environment (Cui et al., 2020). Since mitochondria and its related mitochondrial dynamics are the major therapeutic targets of D1-NBP in various models of diseases, we reasoned that the therapeutic targets of D1-NBP on hypoxic-ischemic demyelination are done by regulating the axonal mitochondrial dynamics in the BCAS mice. By detecting the mitochondrial load among the axons using TEM, we found that the mitochondrial load among the axons was significantly increased in the BCAS group, which was accompanied with abnormal mitochondrial morphology. However, a high-dose treatment of DI-NBP significantly mitigated the mitochondrial load and alleviated the abnormal mitochondrial morphology in the BCAS mice (Figures 2A–D).

Mitochondrial load among the axons was further determined by mitochondrial dynamics and motor proteins that underlie the changes in the mitochondrial dynamics. We probed the protein changes related to mitochondrial dynamics. Although motor proteins, such as Miro and Milton, did not show significant changes after BCAS, SNPH, which anchored the mitochondria to the microtube, showed significant elevation after BCAS. Interestingly, a high-dose treatment of D1-NBP significantly mitigated the SNPH expression, indicating that a high-dose treatment of DI-NBP mitigated mitochondrial accumulation probably by inhibiting the expression of SNPH (Figures 2E,F).

### DI-NBP Alleviates Mitochondrial Accumulation by Promoting Mitochondrial Dynamics

To further clarify the mechanisms underlying the mitigated mitochondrial accumulation by D1-NBP, we established an *in vitro* model of hypoperfusion by supplying chronic LGLO conditions in the culture containing slices of cerebellum. We used lentivirus to overexpress SNPH, and the slices of cerebellum were transfected 72 h before the LGLO treatment. Right after the LGLO treatment, D1-NBP, at a dose of 50 μm, was added to the culture medium and the mitochondrial dynamics were assessed 48 h later.

**Figure 3:**
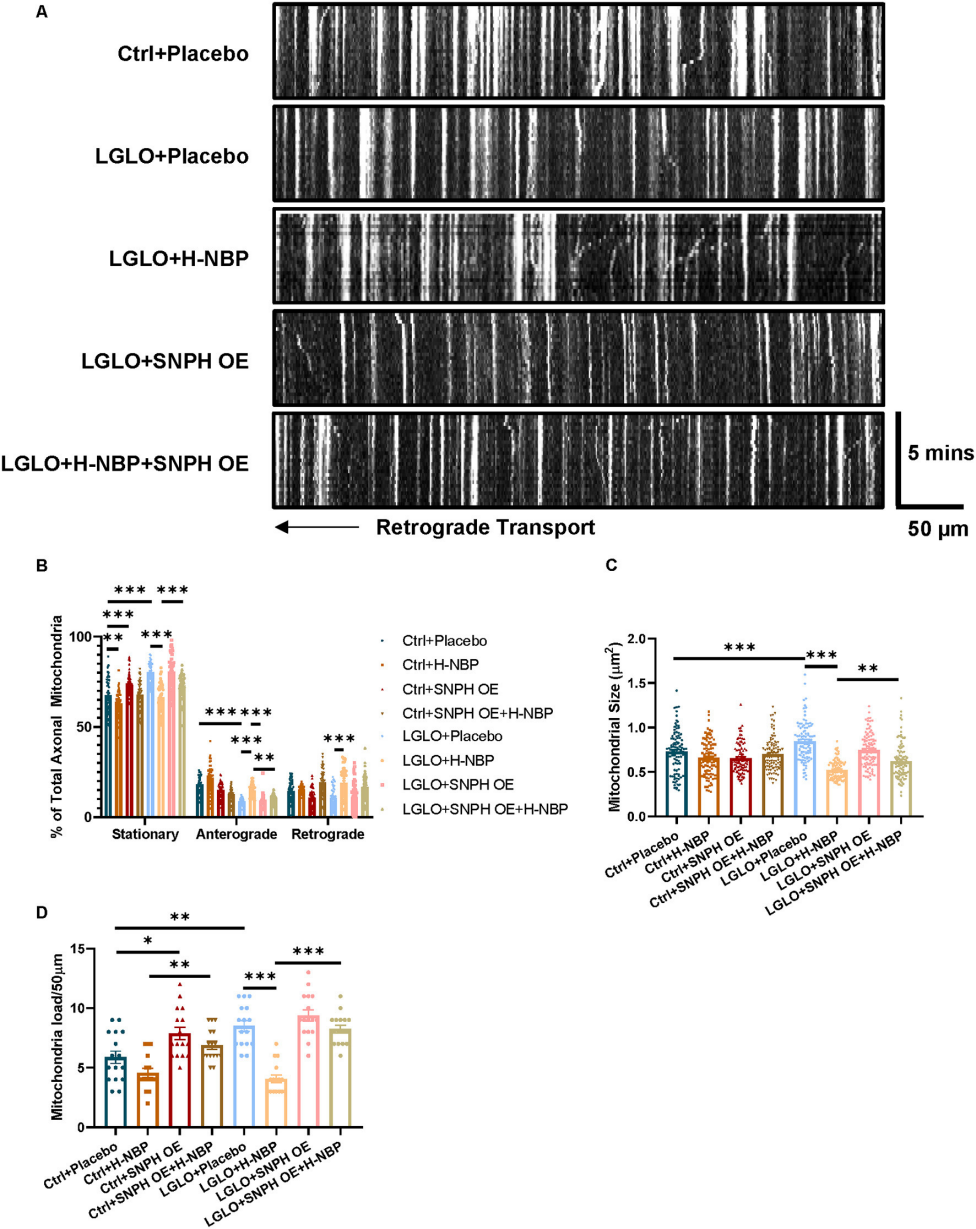
**(A)** High-dose treatment of D1-NBP alleviates mitochondrial accumulation by promoting mitochondrial dynamics. **(A–D)** Representative kymograph **(A)** and quantitative analysis of the percentage of stationary, anterograde, and retrograde mitochondria in the axons **(B)**, mitochondrial load per 50 μm **(C)**, and mitochondrial size **(D)** in cerebellum slices subjected to Ctrl+ phosphate buffered saline (PBS), Ctrl+NBP, Ctrl+SNPH OE, Ctrl+SNPH OE+H-NBP, low glucose and low oxygen (LGLO)+PBS, LGLO+NBP, LGLO+SNPH OE, LGLO+SNPH OE+H-NBP. The high-dose treatment of D1-NBP alleviated the LGLO-induced impairment of retrograde and anterograde mitochondrial transport. A one-way ANOVA with Tukey's correction. For the percentage stationary, anterograde, and retrograde mitochondria analysis, *n* = 60 mitochondria per group. For mitochondrial load per 50 μm analysis, *n* = 20 axons per group. For mitochondrial size analysis, *n* = 400 per group. Scale bar, 50 μm and 5 min, respectively. Data are represented as means ± SEM (^*^p < 0.05; ^**^p < 0.01; ^***^p < 0.001; ns, non-significant differences).

We found that the LGLO treatment significantly increased the mitochondrial load, which was accompanied with decreased mitochondrial dynamics, as evidenced by increased stationary mitochondria after LGLO. Interestingly, the DI-NBP treatment rescued the dynamic drop by LGLO and alleviated the mitochondrial load among the axons. Since we found that a high-dose treatment of DI-NBP mitigates mitochondrial load by inhibiting the expression of SNPH *in vivo*, we further overexpressed the SNPH on the cultured slices and found that the alleviated mitochondrial load by a high dose of DI-NBP was abolished by the SNPH OE (Figures 3A–D).

### Enhanced Mitochondrial Dynamics by DI-NBP Alleviates Demyelination by Decreasing ROS Production Among the Axons

**Figure 4:**
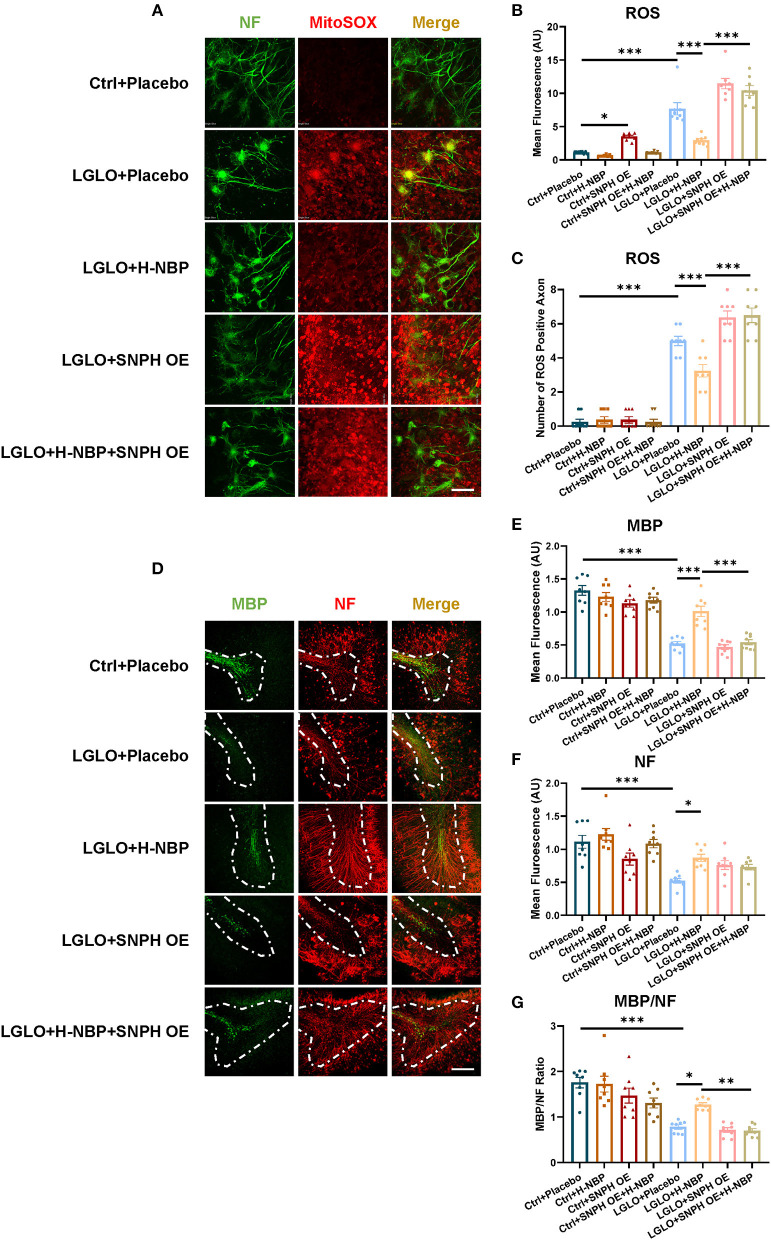
Enhanced retrograde transport of mitochondria by a high-dose treatment of D1-NBP alleviated demyelination by decreasing the ROS production among the axons. **(A–C)** Representative confocal images **(A)** and quantitative analysis of the ROS expression **(B)** and the number of ROS positive axons **(C)**. Scale bar, 100 μm. The high-dose treatment of D1-NBP mitigated LGLO-induced ROS generation. A one-way ANOVA with Tukey's correction, *n* = 8 slices per group. **(D–G)** Representative confocal images **(D)** and quantitative analysis of maltose binding protein (MBP) (green) expression **(E)**, NF (red) expression **(F)**, and the MBP/NF ratio **(G)**. High dose of DI-NBP treatment alleviated LGLO-induced demyelination. Scale bar, 50 μm. A one-way ANOVA with Tukey's correction. *n* = 8 slices per group. Data are represented as means ± SEM (^*^p < 0.05; ^**^p < 0.01; ^***^p < 0.001; ns, non-significant differences).

Functional mitochondrial dynamics plays a critical role in maintaining mitochondrial homeostasis. Impaired transportation of mitochondria and mitochondrial overload in the axons are harmful to neurons, especially in terms of disrupted clearance of malfunctioning mitochondria through retrograde transport (López-Doménech et al., 2018). Increased ROS production has also been reported to damage myelination in the model of multiple sclerosis (MS) (Su et al., 2013). We, therefore, detected mitochondrial ROS production after LGLO. We found that the production of ROS was elevated after LGLO, but the D1-NBP treatment significantly decreased the ROS among axons and mitigated the overall ROS production (Figures 4A–C). Thus, the impaired mitochondrial dynamics were responsible for the mitochondrial accumulation and it significantly increased the production of ROS among the axons. This is harmful for myelination, as evidenced by the significantly decreased MBP expression, NF expression, and MBP/NF ratio in the LGLO group. The DI-NBP mitigated myelination, which is dependent on SNPH (Figures 4D–G).

Together, the DI-NBP treatment decreased the ROS production among axons and rescued myelination, which is related to the improved mitochondria retrograde transport of mitochondria.

### Enhanced Dynamics of Mitochondria by DI-NBP Retrieves Impaired Neuronal Synapse Signaling

**Figure 5:**
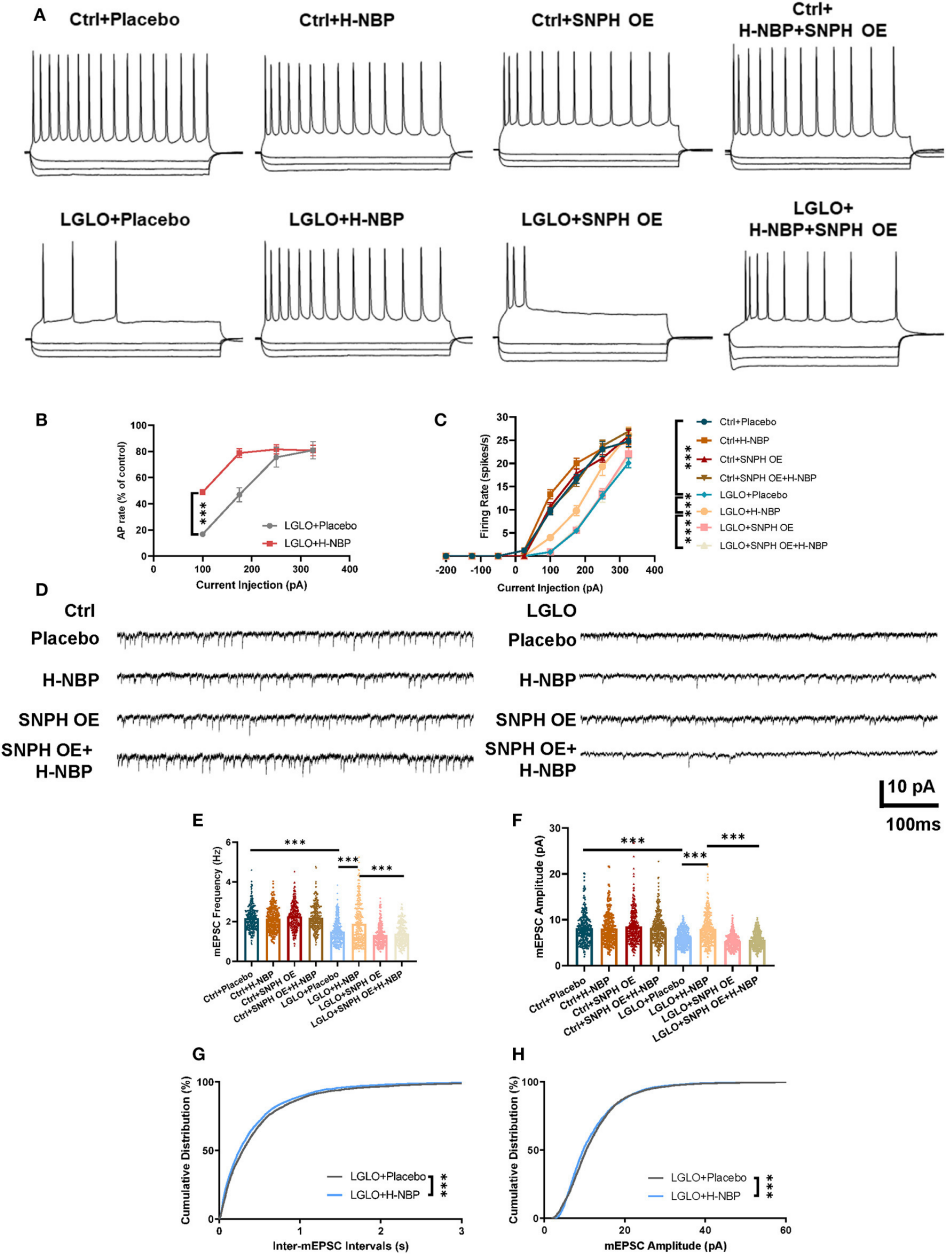
Enhanced anterograde transport of mitochondria by a high-dose treatment of D1-NBP retrieved impaired neuronal synapse signaling. **(A–C)** Representative firing responses **(A)** to depolarizing (175 pA) and hyperpolarizing (−50, −135, and −200 pA) current injections and quantitative analysis percentage of control firing rates and **(B)** firing rate responses to a series of linear current injections (−200 to −50 pA) **(C)** in different treated cerebellum slices. A two-way ANOVA with the Dunnett's *post-hoc* test. For the percentage of the control firing rate analysis, *n* = 9 neurons per group (3 neurons per slices). For firing rate responses to a series of linear current injection analysis, *n* = 9 neurons per group (3 neurons per slices). **(D–H)** Representative mEPSC traces **(D)** and quantitative analysis of miniature excitatory postsynaptic current (mEPSC) frequency **(E)**, amplitude **(F)**, and cumulative distribution of inter-mEPSC interval **(G)** and mEPSCamplitude **(H)**. Under LGLO conditions, impaired intrinsic neuronal excitability and synapse function showed remarkable improvement by H-NBP treatment. For mEPSC frequency and amplitude, one-way ANOVA with Tukey's correction. For cumulative distribution of inter-mEPSC interval and mEPSC amplitude, the Mann–Whitney test. For mEPSC frequency, *n* = 30 per group. For mEPSC amplitude, *n* = 30 per group. For cumulative distribution of inter-mEPSC interval, *n* = 4,291 per group. For cumulative distribution of mEPSC amplitude, *n* = 4,319 per group. Scale bar, 10 pA and 100 ms, respectively. Data are represented as means ± SEM (^***^p < 0.001; ns, non-significant differences).

Anterograde transport of mitochondria replenishes fresh mitochondria necessary for synaptic function (Hollenbeck and Saxton, 2005; Lovas and Wang, 2013). Since we detected an increased anterograde mitochondrial transport, we then tested the changes in the synapse signaling after DI-NBP treatment and under LGLO by patch-clamp. The LGLO treatment resulted in a remarkable drop in firing rate at all injection amplitudes and the most significant drop in firing rate was observed in relatively small current injections. However, the DI-NBP treatment rescued the neuronal intrinsic excitability and SNPH OE abolished the therapeutic effect of D1-NBP to some extent (Figures 5A–C). We further tested the changes in the synaptic signaling after LGLO. Both mEPSC amplitude and frequency decreased dramatically after the LGLO treatment, whereas a high dose of DI-NBP retrieved the drop of mEPSC amplitude and frequency, and SNPH OE abolished these therapeutic effects (Figures 5D–F). A cumulative mEPSC distribution curve analysis showed that the D1-NBP treatment built up a significantly more abundant mEPSC amplitude and smaller inter-mEPSC intervals (Figures 5G,H). These results indicated that D1-NBP promoted the neuronal intrinsic excitability and synapse function, which is related to mitochondrial anterograde transportation.

### DI-NBP Mitigates Demyelination and Cognitive Impairment by Inhibiting SNPH *in vivo*

**Figure 6:**
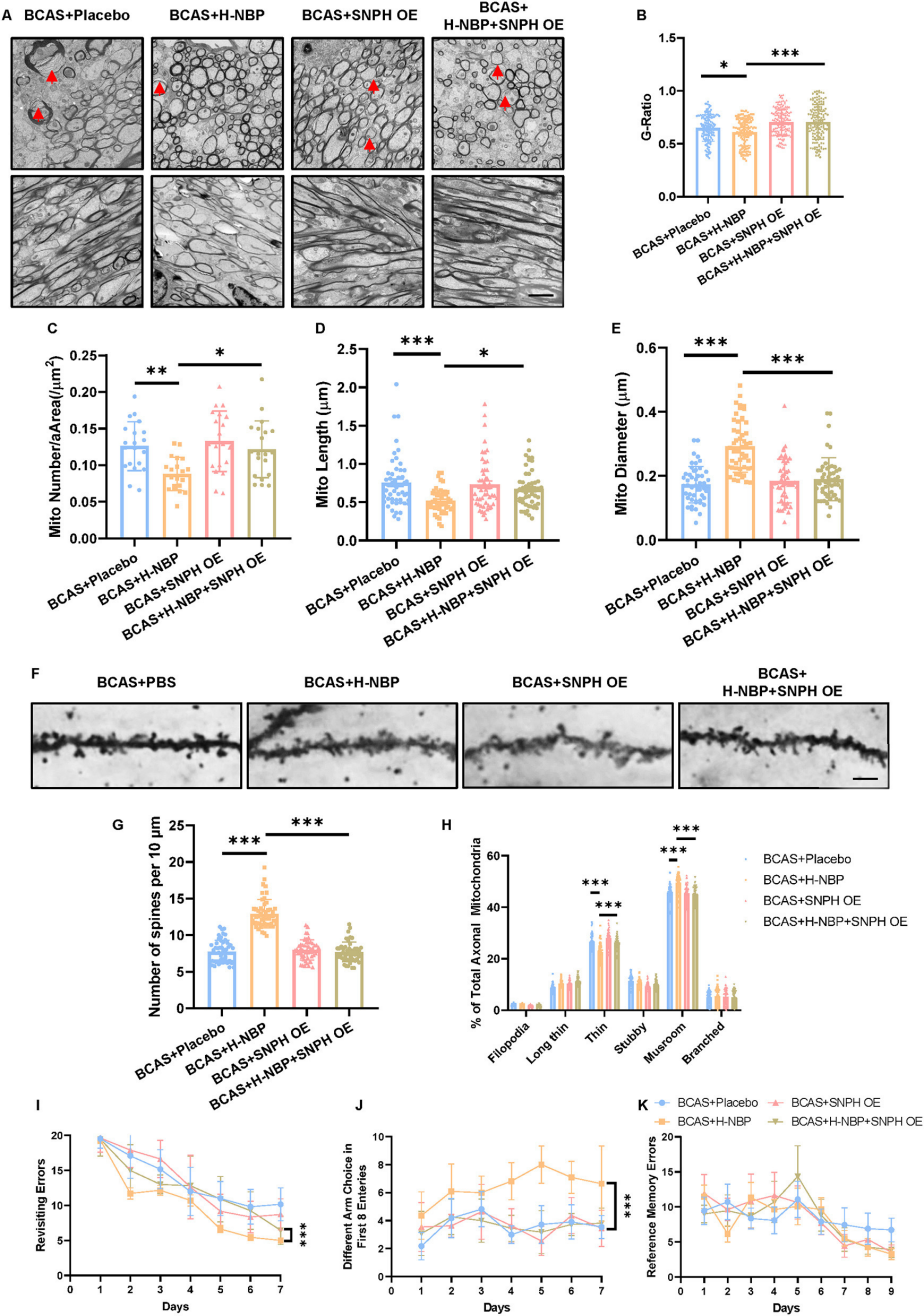
**(A)** High-dose treatment of D1-NBP mitigated demyelination and cognitive impairment by rescuing mitochondrial dynamic *in vivo*. **(A–E)** Representative TEM images depicting myelination status (Upper panel) and mitochondrial load in the axons **(A)** and quantitative analysis of the G-ratio **(B)**, mitochondrial load per area **(C)**, mitochondrial length **(D)**, and mitochondrial diameter **(E)** in BCAS+Placebo, BCAS+H-NBP, BCAS+SNPH OE, and BCAS+SNPH OE+H-NBP groups. The therapeutic effects of H-NBP on mitigating demyelination and cognitive impairment were dependent on mitochondrial dynamics. Scale bar, 2 μm. A one-way ANOVA with Tukey's correction. For G-ratio, *n* = 80 myelinated axons. For mitochondrial load per area analysis, *n* = 20 visual fields (4 visual fields per mouse, 5 mice per group). For mitochondrial length analysis, *n* = 50 (10 mitochondria per mouse, 5 mice per group). For mitochondrial diameter analysis, *n* = 50 (10 mitochondria per mouse, 5 mice per group). **(F–H)** Representative silver staining images. **(F)** Quantitative analysis of the number of spines per 10 μm. **(G)** Different kinds of spine morphology in different groups **(H)**. A one-way ANOVA with Tukey's correction. For the number of spines per 10 μm analysis, *n* = 10 axons per mouse, 5 mice per group. For different kinds of spine morphology analysis, *n* = 10 axons per mouse, 5 mice per group. Scale bar, 10 μm. **(I–K)** Working and reference memory were assessed by the eight-arm radial maze. The improvement of working memory by H-NBP in BCAS mice was abolished by SNPH OE, as evidenced by the increased revisiting errors. **(I)** Less different choices **(J)** in the BCAS+H-NBP+SNPH OE group. There is no significant difference in the spatial reference memory between different groups **(K)**. A two-way ANOVA with the Dunnett's *post-hoc* test, *n* = 11 mice in each group. Data are represented as means ± SD (^*^p < 0.05; ^**^p < 0.01; ^***^p < 0.001; ns, non-significant differences).

Based on our *in vitro* findings that SNPH OE abolished the effects of DI-NBP in promoting mitochondrial dynamics, we further validated whether SNPH underlies the effects of DI-NBP in mitigating demyelination and cognitive impairment in the BCAS model. An AAV 2/9 overexpressing SNPH was stereoscopically injected into lateral ventricles in neonatal mice. A BCAS surgery was performed at 2 months of age. We found that SNPH OE significantly abolished the therapeutic effect of D1-NBP on mitigating demyelination, as indicated by the elevated G-ratio after SNPH OE in the high-dose DI-NBP treatment group (Figures 6A,B). Meanwhile, decreased mitochondrial load and recovered mitochondrial morphology in the high-dose DI-NBP treatment group were also diminished by SNPH OE (Figures 6C–E). Moreover, increased synapse through a high-dose treatment of D1-NBP was lost in the DI-NBP+SNPH OE group, which was accompanied by fewer mushroom-shaped synapses (Figures 6F–H). Improved working memory by DI-NBP was abandoned by SNPH OE (Figures 6I–K).

From the above results, we could draw the conclusion that the therapeutic potential of D1-NBP on mitigating demyelination and cognitive impairment in hypoxic–ischemic demyelination was done by abolishing SNPH elevation among the axons, which retrieved the malfunctioned mitochondrial dynamics and promoted the anterograde mitochondrial transport for synapse signaling and retrograde mitochondrial transport for ROS alleviation by mitophagy.

## Discussion

The results of the present study have demonstrated the effects of Dl-NBP on mitigating demyelination as well as the effects of Dl-NBP in promoting axonal mitochondrial dynamics in WMLs using a BCAS mice model as well as an *in vitro* brain slice culture treated with LGLO. Our results demonstrated that the mitochondrial dynamics were suppressed while the static mitochondria accumulated in the axons after an injury to ischemia/hypoxia, which was mediated by the elevated expression of SNPH, an axonal specific arching protein. A high-dose treatment of Dl-NBP improved the anterograde and retrograde transport of mitochondria and reduced mitochondrial accumulation in axons, thus arresting myelin disruption and improving the electrical function of the synapse. The high-dose treatment of Dl-NBP also improved the number of synapses and cognitive function. In addition, we investigated the mechanism of Dl-NBP and revealed that a high-dose treatment of Dl-NBP suppressed the expression of SNPH. SNPH OE abolished the protective function of Dl-NBP in reducing demyelination and improving the cognitive function.

White matter lesions, also termed leukoaraiosis, are very common and are considered as an important contributor to cognitive decline, especially in the elderly (Alber et al., 2019). The primary cerebrovascular pathologies that cause WMLs include multi-arteriolosclerosis, carotid stenosis, or occlusion, which lead to cerebral hypoperfusion in deep white matter regions (van Norden et al., 2011). White matter, composed of bundles of myelinated axons, plays a vital role in signal transmission. It has been reported that the Dl-NBP treatment improved the learning and memory deficits induced by chronic cerebral hypoperfusion in the animal model (Li et al., 2019). We speculate that Dl-NBP improves cognitive function in hypoperfusion partly by preventing the disruption of myelin and increasing the signal transmission. In the present study, we also observed that the Dl-NBP treatment improved the cognitive impairment in the BCAS model. Next, we examined the effects of Dl-NBP on demyelination because the intact and functional myelin is the structural foundation of the electrical signal traveling from the body of the cell down the synapse of the axon. Results showed that a high-dose treatment of Dl-NBP significantly mitigated demyelination compared with the BCAS model group. Further, we evaluated the synapse signaling, which is related to the activity of the central cholinergic system. Results indicated that a high dose of Dl-NBP promoted neuronal intrinsic excitability and synapse function compared with the BCAS model group. These two neuroprotective aspects of Dl-NBP eventually benefited the cognitive function recovery after BCAS.

White matter is more susceptible to chronic cerebral hypoperfusion than gray matter, which involves both axonal and myelin components (Wakita et al., 2002). Myelin is produced by oligodendrocytes, which are attached to the axons and, therefore, could interact with the axons in the central nervous system. During the process of demyelination caused by hypoperfusion, the axonal changes are non-ignorable. It was reported recently that the degradation of axons was accompanied by mitochondrial shortening in the *in vitro* model of WML (Cui et al., 2020). While in our study, we observed abnormal-shaped mitochondria in the BCAS mice showed an increased length and a reduced diameter. After a high-dose treatment of Dl-NBP, the morphological changes of mitochondria recovered significantly. Mitochondria are the most sensitive organisms to hypoxia and respond quickly upon ischemial insult. Normally, mitochondria undergo massive fusion and fission events, as well as transportation along the axons, to continuously maintain their function and maintain the energy supply to cells (Youle and van der Bliek, 2012; Lee and Yoon, 2016; Meyer et al., 2017). An impaired balance of mitochondrial dynamics occurs under hypoperfusion. Meanwhile, we observed an increased number of abnormal mitochondria accumulated in the axons of BCAS mice, but a high-dose treatment of Dl-NBP reduced their accumulation. These results indicated that Dl-NBP targets axonal mitochondria in alleviating demyelination damage.

Syntaphilin is a major mitochondrial anchoring protein targeting the axons (Joshi et al., 2019). The SNPH deletion produces striking benefits in the MS demyelination model by prolonging survival, reducing cerebellar damage, suppressing oxidative stress, and improving mitochondrial health (Joshi et al., 2015). Later, we examined the SNPH levels and found a high expression of SNPH in BCAS mice, accompanied with increased stationary mitochondria in the axons. A high-dose treatment of Dl-NBP inhibited the SNPH levels, which then promoted both the anterograde and retrograde transport of mitochondria. Through SNPH OE, the protective effects of Dl-NBP on mitigating demyelination disappeared. Further, the protection of cognitive function was abolished as well. Thus, we found SNPH to be a novel target of Dl-NBP in hypoperfusion-induced WML.

One of the primary roles of mitochondria is to produce ATP. The anterograde transport of mitochondria will increase mitochondrial respiration and ATP production (Roger et al., 2017; Rangaraju et al., 2019). The increased anterograde transport of mitochondria might help the axons to maintain energy stability, and this is likely to underlie the improved synapse signaling function in a high-dose Dl-NBP treated group. The retrograde transport of mitochondria causes mitophagy of dysfunctional mitochondria, which alleviated the ROS production. Since myelin is sensitive to ROS, the reduced ROS production in Dl-NBP treated mice may explain the protection of myelin and keep the structure of the synapses intact.

In conclusion, our results indicate that a high dose of Dl-NBP inhibited the expression of SNPH, which is an axonal specific mitochondrial arching protein. The SNPH inhibition by Dl-NBP alleviates mitochondrial load among the axons and promotes the anterograde and retrograde mitochondrial transport, which then rescued demyelination and cognitive function in the WML model. These findings of our study suggest that Dl-NBP is a promising treatment for alleviating the cognitive dysfunction and alleviating demyelination in WML.

## Data Availability Statement

The original contributions presented in the study are included in the article/supplementary material, further inquiries can be directed to the corresponding authors.

## Ethics Statement

The animal study was reviewed and approved by the Ethics Committee of Fudan University, Shanghai, China. Written informed consent was obtained from the owners for the participation of their animals in this study.

## Author Contributions

YWF and MG drafted the manuscript. YWF and SH carried out the experiment. YWF and HZ helped with the statistics and the preparation of figures. MC and QD designed the experiment. All authors read and approved the final manuscript.

## Conflict of Interest

The authors declare that the research was conducted in the absence of any commercial or financial relationships that could be construed as a potential conflict of interest.

## Abbreviations

WMLs: white matter lesions
APs: action potentials
BCAS: bilateral common carotid artery stenosis
LGLO: low glucose and low oxygen
mEPSC: miniature excitatory postsynaptic current
MS: multiple sclerosis
SNPH: syntaphilin
TEM: transmission electron microscopy
VaD: vascular dementia.

## References

[B1] Alber J. Alladi S. Bae H. J. Barton D. A. Beckett L. A. Bell J. M.. (2019). White matter hyperintensities in vascular contributions to cognitive impairment and dementia (VCID): knowledge gaps and opportunities. Alzheimers Dement 5, 107–117. 10.1016/j.trci.2019.02.001 31011621 PMC6461571

[B2] Bargiela D. Burr S. P. Chinnery P. F. (2018). Mitochondria and hypoxia: metabolic crosstalk in cell-fate decisions. Trends Endocrinol. Metab. 29, 249–259. 10.1016/j.tem.2018.02.002 29501229

[B3] Ben-Ari H. Lifschytz T. Wolf G. Rigbi A. Blumenfeld-Katzir T. Merzel T. K.. (2019). White matter lesions, cerebral inflammation and cognitive function in a mouse model of cerebral hypoperfusion. Brain Res. 1711, 193–201. 10.1016/j.brainres.2019.01.017 30659829

[B4] Chen D. Yin Y. Shi J. Yang F. Wang K. Zhao F.. (2020). DL-3-n-butylphthalide improves cerebral hypoperfusion in patients with large cerebral atherosclerotic stenosis: a single-center, randomized, double-blind, placebo-controlled study. BMC Neurol. 20:212. 10.1186/s12883-020-01801-5 32456617 PMC7251861

[B5] Chen J. Liu N. Wang X. Zhao Y. He J. Yang L.. (2019). Dl-3-n-butylphthalide inhibits phenytoin-induced neuronal apoptosis in rat hippocampus and cerebellum. J. Integr. Neurosci. 18, 277–283. 10.31083/j.jin.2019.03.174 31601076

[B6] Chen N. Zhou Z. Li J. Li B. Feng J. He D.. (2018). 3-n-butylphthalide exerts neuroprotective effects by enhancing anti-oxidation and attenuating mitochondrial dysfunction in an *in vitro* model of ischemic stroke. Drug Des. Devel. Ther. 12, 4261–4271. 10.2147/DDDT.S189472 30587922 PMC6298396

[B7] Cui L. Y. Zhu Y. C. Gao S. Wang J. M. Peng B. Ni J.. (2013). Ninety-day administration of dl-3-n-butylphthalide for acute ischemic stroke: a randomized, double-blind trial. Chin. Med. J. 126, 3405–3410. 10.3760/cma.j.issn.0366-6999.20123240 24034079

[B8] Cui Y. Jin X. Choi D. J. Choi J. Y. Kim H. S. Hwang D. H.. (2020). Axonal degeneration in an *in vitro* model of ischemic white matter injury. Neurobiol. Dis. 134:104672. 10.1016/j.nbd.2019.104672 31707117

[B9] Datta G. Colasanti A. Rabiner E. A. Gunn R. N. Malik O. Ciccarelli O.. (2017). Neuroinflammation and its relationship to changes in brain volume and white matter lesions in multiple sclerosis. Brain 140, 2927–2938. 10.1093/brain/awx228 29053775

[B10] Du F. (2019). Golgi-cox staining of neuronal dendrites and dendritic spines with FD Rapid GolgiStain™ Kit. Curr. Protoc. Neurosci. 88:e69. 10.1002/cpns.69 31216393

[B11] Feng Y. W. Huang Y. Q. Yan Y. Li G. He X. F. Liang F. Y.. (2020a). Phasic GABA signaling mediates the protective effects of cTBS against cerebral ischemia in mice. Neurosci. Lett. 715:134611. 10.1016/j.neulet.2019.134611 31698026

[B12] Feng Y. W. Wu C. Liang F. Y. Lin T. Li W. Q. Jing Y. H.. (2020b). hUCMSCs mitigate LPS-induced trained immunity in ischemic stroke. Front. Immunol. 11:1746. 10.3389/fimmu.2020.01746 33013828 PMC7516337

[B13] Guo M. Ma X. Feng Y. Han S. Dong Q. Cui M.. (2019). In chronic hypoxia, glucose availability and hypoxic severity dictate the balance between HIF-1 and HIF-2 in astrocytes. FASEB J. 33, 11123–11136. 10.1096/fj.201900402RR 31298941

[B14] Hollenbeck P. J. Saxton W. M. (2005). The axonal transport of mitochondria. J. Cell Sci. 118(Pt 23), 5411–5419. 10.1242/jcs.02745 16306220 PMC1533994

[B15] Joshi D. C. Zhang C. L. Babujee L. Vevea J. D. August B. K. Sheng Z. H.. (2019). Inappropriate intrusion of an axonal mitochondrial anchor into dendrites causes neurodegeneration. Cell Rep. 29, 685–696.e5. 10.1016/j.celrep.2019.09.012 31618636 PMC6884150

[B16] Joshi D. C. Zhang C. L. Lin T. M. Gusain A. Harris M. G. Tree E.. (2015). Deletion of mitochondrial anchoring protects dysmyelinating shiverer: implications for progressive MS. J. Neurosci. 35, 5293–5306. 10.1523/JNEUROSCI.3859-14.2015 25834054 PMC4381002

[B17] Lee H. Yoon Y. (2016). Mitochondrial fission and fusion. Biochem. Soc. Trans. 44, 1725–1735. 10.1042/BST20160129 27913683

[B18] Lema A. Bishop C. Malik O. Mattoscio M. Ali R. Nicholas R.. (2017). A comparison of magnetization transfer methods to assess brain and cervical cord microstructure in multiple sclerosis. J. Neuroimaging 27, 221–226. 10.1111/jon.12377 27491693

[B19] Li W. Wei D. Lin J. Liang J. Xie X. Song K.. (2019). Dl-3-n-butylphthalide reduces cognitive impairment induced by chronic cerebral hypoperfusion through GDNF/GFRα1/Ret signaling preventing hippocampal neuron apoptosis. Front. Cell. Neurosci. 13:351. 10.3389/fncel.2019.00351 31456664 PMC6701226

[B20] Lin M. Y. Cheng X. T. Tammineni P. Xie Y. Zhou B. Cai Q.. (2017). Releasing syntaphilin removes stressed mitochondria from axons independent of mitophagy under pathophysiological conditions. Neuron. 94, 595–610.e6. 10.1016/j.neuron.2017.04.004 28472658 PMC5484086

[B21] López-Doménech G. Covill-Cooke C. Ivankovic D. Halff E. F. Sheehan D. F. Norkett R.. (2018). Miro proteins coordinate microtubule- and actin-dependent mitochondrial transport and distribution. EMBO J. 37, 321–336. 10.15252/embj.201696380 29311115 PMC5793800

[B22] Lovas J. R. Wang X. (2013). The meaning of mitochondrial movement to a neuron's life. Biochim. Biophys. Acta 1833, 184–194. 10.1016/j.bbamcr.2012.04.007 22548961 PMC3413748

[B23] Meyer J. N. Leuthner T. C. Luz A. L. (2017). Mitochondrial fusion, fission, and mitochondrial toxicity. Toxicology 391, 42–53. 10.1016/j.tox.2017.07.019 28789970 PMC5681418

[B24] Palikaras K. Lionaki E. Tavernarakis N. (2015). Coordination of mitophagy and mitochondrial biogenesis during ageing in C. elegans. Nature 521, 525–528. 10.1038/nature14300 25896323

[B25] Rangaraju V. Lewis T. L. Jr. Hirabayashi Y. Bergami M. Motori E. Cartoni R.. (2019). Pleiotropic mitochondria: the influence of mitochondria on neuronal development and disease. J. Neurosci. 39, 8200–8208. 10.1523/JNEUROSCI.1157-19.2019 31619488 PMC6794931

[B26] Roger A. J. Muñoz-Gómez S. A. Kamikawa R. (2017). The origin and diversification of mitochondria. Curr. Biol. 27, r1177–r1192. 10.1016/j.cub.2017.09.015 29112874

[B27] Su K. Bourdette D. Forte M. (2013). Mitochondrial dysfunction and neurodegeneration in multiple sclerosis. Front. Physiol. 4:169. 10.3389/fphys.2013.00169 23898299 PMC3722885

[B28] Sun Y. Cheng X. Wang H. Mu X. Liang Y. Luo Y.. (2017). dl-3-n-butylphthalide promotes neuroplasticity and motor recovery in stroke rats. Behav. Brain Res. 329, 67–74. 10.1016/j.bbr.2017.04.039 28442357

[B29] Thomas L. W. Ashcroft M. (2019). Exploring the molecular interface between hypoxia-inducible factor signalling and mitochondria. Cell. Mol. Life Sci. 76, 1759–1777. 10.1007/s00018-019-03039-y 30767037 PMC6453877

[B30] Tian A. Li W. Zai Q. Li H. Zhang R. W. (2020). 3-N-Butyphthalide improves learning and memory in rats with vascular cognitive impairment by activating the SIRT1/BDNF pathway. Mol. Med. Rep. 22, 525–533. 10.3892/mmr.2020.11106 32377741 PMC7248482

[B31] van Norden A. G. de Laat K. F. Gons R. A. van Uden I. W. van Dijk E. J. van Oudheusden L. J.. (2011). Causes and consequences of cerebral small vessel disease. The RUN DMC study: a prospective cohort study. Study rationale and protocol. BMC Neurol. 11:29. 10.1186/1471-2377-11-29 21356112 PMC3053228

[B32] Wakita H. Tomimoto H. Akiguchi I. Matsuo A. Lin J. X. Ihara M.. (2002). Axonal damage and demyelination in the white matter after chronic cerebral hypoperfusion in the rat. Brain Res. 924, 63–70. 10.1016/S0006-8993(01)03223-1 11743996

[B33] Wang C. Y. Xu Y. Wang X. Guo C. Wang T. Wang Z. Y. (2019). Dl-3-n-butylphthalide inhibits NLRP3 inflammasome and mitigates Alzheimer's-like pathology via Nrf2-TXNIP-TrX axis. Antioxid. Redox Signal. 30, 1411–1431. 10.1089/ars.2017.7440 29634349

[B34] Wang S. Ma F. Huang L. Zhang Y. Peng Y. Xing C.. (2018). Dl-3-n-butylphthalide (NBP): a promising therapeutic agent for ischemic stroke. CNS Neurol. Disord. Drug Targets 17, 338–347. 10.2174/1871527317666180612125843 29895257

[B35] Xu H. Baracskay P. O'Neill J. Csicsvari J. (2019). Assembly responses of hippocampal CA1 place cells predict learned behavior in goal-directed spatial tasks on the radial eight-arm maze. Neuron 101, 119–132.e4. 10.1016/j.neuron.2018.11.015 30503645

[B36] Xue L.-X. Zhang T. Zhao Y.-W. Geng Z. Chen J.-J. Chen H. (2016). Efficacy and safety comparison of DL-3-n-butylphthalide and Cerebrolysin: Effects on neurological and behavioral outcomes in acute ischemic stroke. Exp. Ther. Med. 11, 2015–2020. 10.3892/etm.2016.3139 27168844 PMC4840538

[B37] Yang C. S. Guo A. Li Y. Shi K. Shi F. D. Li M. (2019). Dl-3-n-butylphthalide reduces neurovascular inflammation and ischemic brain injury in mice. Aging Dis. 10, 964–976. 10.14336/AD.2019.0608 31595195 PMC6764730

[B38] Youle R. J. van der Bliek A. M. (2012). Mitochondrial fission, fusion, and stress. Science 337, 1062–1065. 10.1126/science.1219855 22936770 PMC4762028

[B39] Zheng Y. Wu X. Chen Z. Zhang X. (2019). Come and eat: mitochondrial transport guides mitophagy in ischemic neuronal axons. Autophagy 15, 1483–1484. 10.1080/15548627.2019.1618099 31095452 PMC6613904

